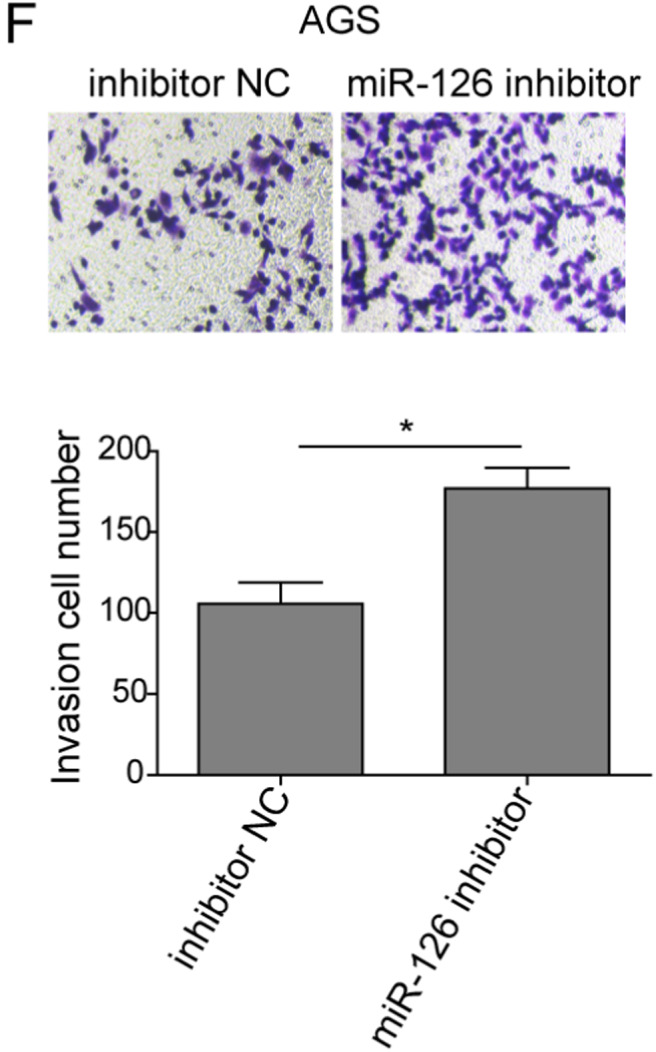# Corrigendum

**DOI:** 10.1002/cam4.4435

**Published:** 2021-11-22

In the article by Xiao et al.,^1^ entitled “lncRNA HOTAIR promotes gastric cancer proliferation and metastasis via targeting miR‐126 to active CXCR4 and RhoA signaling pathway,” the author wants to correct figure parts 2D, 3D, 5C, and 5F.

Corrected Figure 2D.

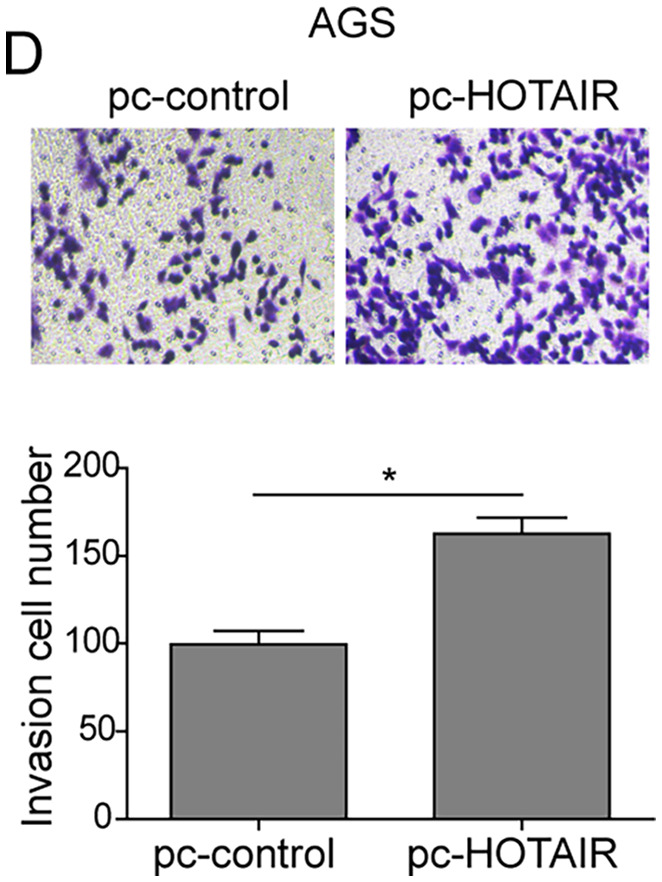

Corrected Figure 3D.

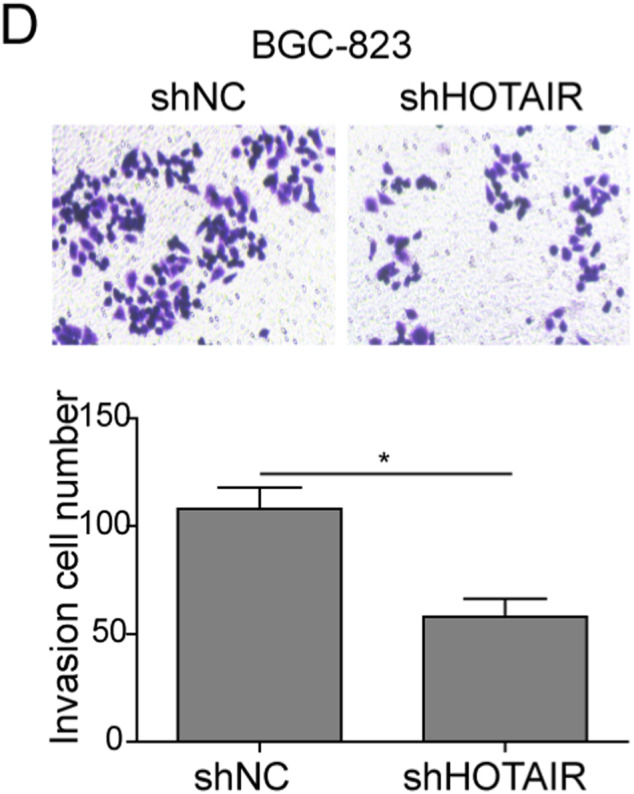

Corrected Figure 5C.

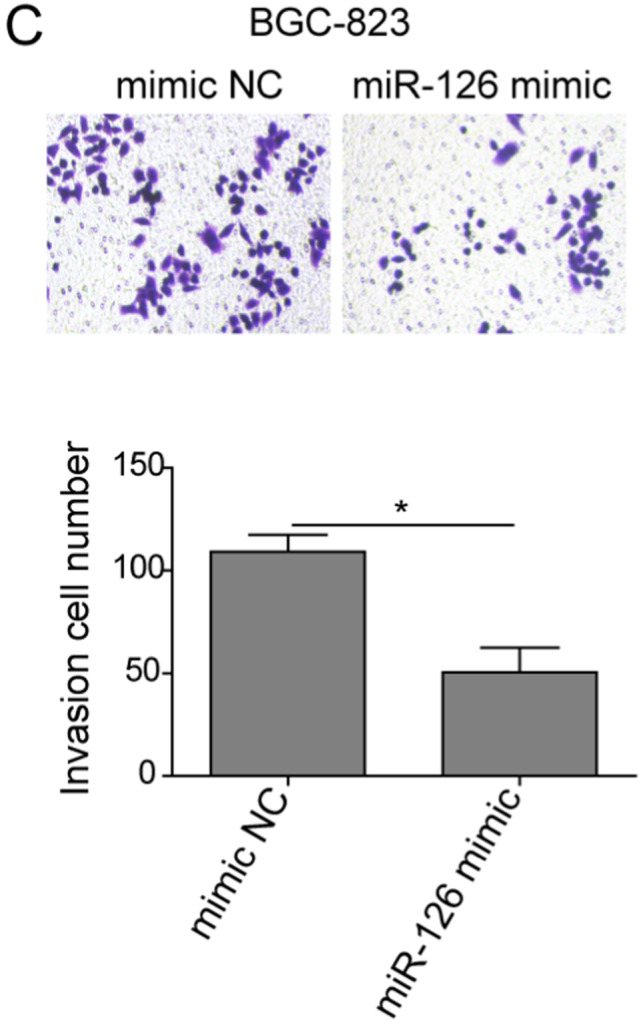

Corrected Figure 5F.